# Decreased incidence of SIRS and sepsis by acupuncture in severe multiple traumatic patients via facilitation of vagal activity

**DOI:** 10.1186/cc11725

**Published:** 2012-11-14

**Authors:** H Liang, J Qu

**Affiliations:** 1 Research Institute of Surgery, Daping Hospital, Daping, Chongqing, China

## Background

Acupuncture could increase vagal activity. We hypothesize that acupuncture could activate the cholinergic anti-inflammatory pathway, inhibit systemic inflammation, and improve the clinical prognosis in severe multiple trauma patients.

## Methods

The design was a prospective, randomized intervention trial. The setting was the Trauma Center, Department of Emergency Medicine, Southwest Hospital, Chongqing, P.R. China. Consecutive multiple trauma patients with an Injury Severity Score (ISS) of more than 16 points meeting the criteria between January 2007 and December 2008 were included. The intervention acupuncture was applied to the ST-36 and PC-6 acupoint twice a day until day 7 after injury or sham acupuncture was applied to non-acupoints. One hundred and fifty severe multiple traumatic patients were randomly divided into three groups: acupuncture group (group A; *n *= 50), sham acupuncture group (group B; *n *= 50), and control group (group C; *n *= 50).

## Results

Acupuncture at ST-36 and PC-6 acupoints significantly increased the normalized unit of the high-frequency component (HFnu; *P *< 0.01) and decreased the ratio of low-frequency and high-frequency component (LF/HF; *P *< 0.05), which indicated an increased vagal activity and a tilt of the sympathovagal balance. Serum TNFα and IL-6 concentrations significantly decreased after stimulation at ST-36 and PC-6 acupoints (*P *< 0.01 and *P *< 0.05, respectively), but remained unchanged following stimulation at the non-acupoint area. During the whole study period, much lower concentrations of TNFα and IL-6 were detected in severe traumatic patients with acupuncture intervention in comparison with those in sham acupuncture and control patients (*P *< 0.05). Most importantly, we observed that the incidences of SIRS, and some clinical complications, such as ARDS, sepsis or MOF, and mortality in the acupuncture group were much lower in comparison with the other two groups.

## Conclusion

Acupuncture has beneficial effects on modulation of inflammatory response and will improve the clinical outcomes in severe multiple traumatic patients. We suggest that larger cohort studies or multiple center studies are needed to validate whether acupuncture is another effective stimulation pattern to activate the cholinergic anti-inflammatory pathway. The detailed mechanisms are worth further investigation.

